# New microarchitectures of (Er,Yb):Lu_2_O_3_ nanocrystals embedded in PMMA: synthesis, structural characterization, and luminescent properties

**DOI:** 10.1186/1556-276X-8-385

**Published:** 2013-09-13

**Authors:** Montserrat Galceran, Maria Cinta Pujol, Joan Josep Carvajal, Xavier Mateos, Pilar Formentín, Josep Pallarès, Lluis Francesc Marsal, Kyung Ho Park, Fabian Rotermund, Kihong Kim, Magdalena Aguiló, Francesc Díaz

**Affiliations:** 1 Física i Cristal lografia de Materials i Nanomaterials (FiCMA-FiCNA)-EMAS, Universitat Rovira i Virgili (URV), Campus Sescelades, c/ Marcel lí Domingo, s/n, Tarragona E-43007, Spain; 2 Departament d’Enginyeria Electrònica, Nanoelectronic and Photonic Systems (NePHoS), EMAS, Elèctrica i Autonomatica, ETSE, Universitat Rovira i Virgili (URV), Campus Sescelades, Avda. Paisos Catalans 26, Tarragona E-43007, Spain; 3 Korea Advanced Nano Fab Center (KANC), Suwon 443-270, Republic of Korea; 4 Division of Energy Systems Research, Ajou University, San 5 Wonchun, Suwon 443-749, Republic of Korea

**Keywords:** Hybrid composite, Polymer, Nanocrystals, Sesquioxide, Erbium, Ytterbium

## Abstract

We report the formation of two-dimensional disordered arrays of poly(methyl)methacrylate (PMMA) microcolumns with embedded single size distribution of Lu_0.990_Er_0.520_Yb_0_._490_ nanocrystals, (Er,Yb):Lu_2_O_3_, using a disordered porous silicon template. The cubic (Er,Yb):Lu_2_O_3_ nanocrystals, which crystallize into the cubic system with

$$\mathrm{Ia}\bar{3}$$

space group, were synthesized using the modified Pechini method. Electronic microscopic techniques were used to study the distribution of the nanocrystals in the PMMA columns. Cathodoluminescence was used to observe the visible luminescence of the particles. Red emission attributed to ^4^ F_9/2_ → ^4^I_15/2_ erbium transition is predominant in these new composites.

## Background

In recent years, low-dimensional nanostructured materials based on porous templates have attracted considerable attention due to their potential applications in various kinds of functional devices [1-4]. Depositing specific materials onto porous templates creates ordered or disordered structures with suitable dimensions and periodicity and inverse replicas of the pores, thus allowing the expansion of these materials' possible application.

Porous silicon was discovered by Uhlir (in 1956) and was intensively investigated because of its excellent mechanical and thermal properties [5], its obvious compatibility with silicon-based microelectronics, and its low-cost fabrication [6]. It was found to be a very promising and attractive candidate for use as a template because it can be fabricated with high precision and uniformity on a large scale. The porosity and average pore size and depth can be tuned by adjusting the electrochemical preparation techniques [7-10]. Depositing specific materials, such as polymers and nonlinear materials, into porous templates allows new structures to be tailored [11]. Organic materials such as polymers are favored in many applications because many of these are optically transparent, biocompatible, and/or biodegradable. In addition, polymer devices are inexpensive and disposable. The air holes of porous silicon structures can be infiltrated with these advantageous polymers.

Nanocrystalline materials are generally defined as crystalline solids with grain sizes below 100 nm. The study and synthesis of nanocrystalline materials have been major research interests in recent years due to expectations of finding new or improved optical, electronic, and structural properties related to the nanoscale of materials [12].

The Pechini method is an alternative to the conventional sol–gel method for synthesizing nanocrystals. This chemical route is highly feasible and offers several advantages over conventional techniques, such as lower temperature requirements, lower cost, and greater simplicity [13].

One goal of our research is to make erbium-doped materials that emit light. As a host for erbium, the cubic RE_2_O_3_ (rare earths) are known as excellent optical materials because of their optimal thermal and spectroscopic properties [14]. Efficiency in erbium emissions can be improved by co-doping with ytterbium, thus assuring a high absorption at 980 nm, where high-power diode lasers are commercially available. This class of composite materials has already been reported for planar optical amplifiers [15]. Furthermore, the Er-Yb couple is well known for its up-conversion mechanisms, converting infrared (IR) light o visible light [16]. The green and red emissions achieved by excitation in IR light or higher energies in erbium samples open up the possibility of using these composites as up-converters or down-converters for both solar cell and lighting applications.

In the present work, we describe a new template-based method for fabricating polymeric micro- and nanostructures. This method entails vacuum infiltration of a poly(methyl)methacrylate (PMMA) solution with embedded luminescent nanocrystals into the pores of a silicon template. The nanocrystals have been synthesized using the modified Pechini method. This method should be applicable to any polymer that can be dissolved in a solvent that is compatible with these template membranes.

## Methods

### Synthesis of nanocrystals

(Er,Yb):Lu_2_O_3_ nanocrystals were synthesized using the modified Pechini method, as described in our previous studies [17,18]. The starting materials were Er_2_O_3_ (99.9%; Sigma-Aldrich Corporation, St. Louis, MO, USA), Yb_2_O_3_ (99.999%, Sigma-Aldrich Corporation) and Lu_2_O_3_ (99.9999%, METALL Rare Earth Limited, Shenzhen, China), and these were mixed to obtain stoichiometric products of 25 at.% Er and 25 at.% Yb:Lu_2_O_3_. To synthesize the nanocrystals, rare-earth oxides were first converted to nitrates by dissolving them with HNO_3_ (65%; Merck AG, Darmstadt, Germany) under stirring and heating. Ethylenediaminetetraacetic acid (EDTA) was then added, taking into account the molar ratio *C*_M_ = (EDTA / Metal) = 1, and a solution of metal-EDTA complexes was obtained. Ethylene glycol (EG) was subsequently added to the solution with a molar ratio of *C*_E_ = (EDTA / EG) = 2, and the precursor resin was formed through the esterification reaction while the solution was heated to about 363 K. Finally, the viscous gel obtained was calcinated at 1,073 K in air atmosphere to obtain the (Er,Yb):Lu_2_O_3_ nanocrystals. The *C*_M_ ratio, *C*_E_ ratio, and calcination temperature were already optimized in a previous study.

### Synthesis of PMMA microcolumns

**Figure 1:**
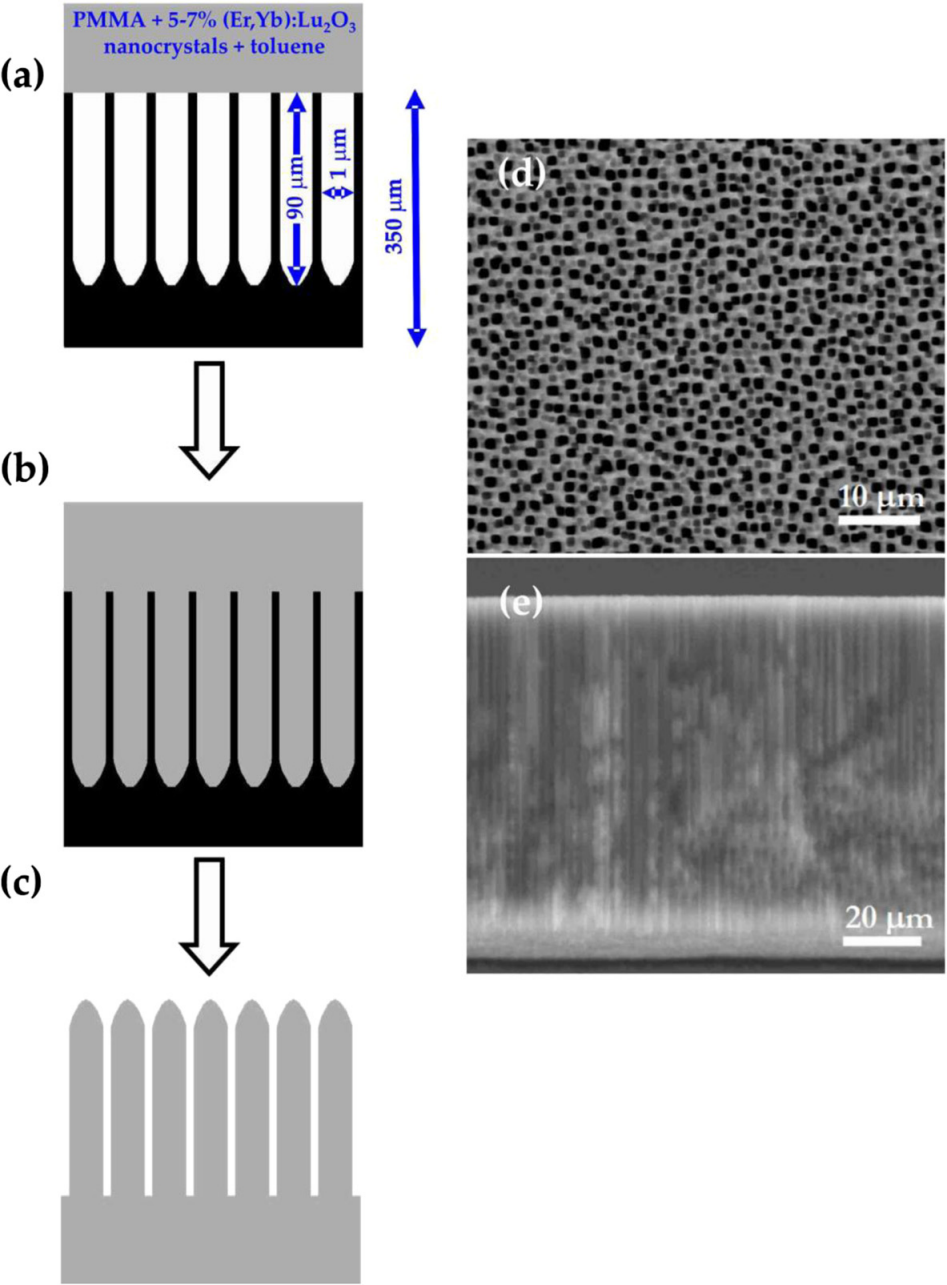
**Schematic diagram of the experimental procedure and photographs of the silicon template. (a, b, c)** Schematic diagram of the experimental procedure for obtaining microcolumns using a disordered silicon template. Photographs of the silicon template: **(d)** general top view and **(e)** cross section.

Macroporous silicon template was prepared by electrochemical etching of p-type silicon wafers with a resistivity of 10 to 20 Ω cm in a mixed solution of HF/DMF (1:10; hydrofluoric acid/dimethylformamide) at room temperature with a current density of 10 mA/cm^2^[19,20]. Figure 1d,e shows the macroporous silicon template obtained with a pore diameter of approximately 1 μm and pore depth of 90 μm. Polymer microcolumns using silicon templates were fabricated by vacuum infiltration of 5 to 7wt.% of (Er,Yb):Lu_2_O_3_ nanocrystals embedded in 15 wt.% poly(methyl) methacrylate in toluene. The technique was an infiltration by putting a drop of the solution on top of the sample located under vacuum (Figure 1a,b,c). The samples were heated at 383 K for 3 h, followed by immersion into 40-wt.% KOH (2 M) at 40°C in order to remove the silicon template [21].

### Characterization techniques

X-ray diffraction measurements were performed using a Bruker-AXS D8-Discover (Karlsruhe, Germany) diffractometer with a parallel incident beam (Göbel mirror) and a vertical goniometer, with a 0.02° receiving slit and a scintillation counter as a detector. A 30 × 30 cm^2^ general area diffraction system with a 1,024 × 1,024 pixel CCD detector was used for the micro-X-ray diffraction (μXRD) analysis, and an X-ray collimator system was used to analyze areas of 500 μm. CuK*α* radiation was obtained from a copper X-ray tube operated at 40 kV and 40 mA. Data were collected with an angular step of 0.02° at 900 s/frame per step. FULLPROF software based on the Rietveld method was used to refine the unit cell parameters [22,23]. The particle size was estimated using Scherrer's equation and assuming spherical particles [24].

The chemical composition of the nanocrystals was examined by electron probe microanalysis (EPMA) in a Cameca SX50 (Gennevilliers Cedex, France) microprobe analyzer operating in wavelength-dispersive mode. The contents of erbium, ytterbium, and lutetium were measured using Lα and LiF as analyzing crystals.

A FEI QUANTA 600 (Hillsboro, OR, USA) environmental scanning electronic microscope (ESEM) and a JEOL JEM-1011 transmission electron microscope (TEM) with MegaView III (Soft Imaging System, Olympus, Tokyo, Japan) were used to study particle homogeneity, morphology, and size dispersion. To examine the samples by TEM, the nanocrystals were dispersed in acetone. Ultrasonication was used to reduce and disperse the agglomerates. They were then drop-cast onto a copper grid covered by a porous carbon film.

Cathodoluminescence (CL) experiments were performed at room temperature using Gatan MonoCL3+ system attached on Schottky-type field-emission scanning electron microscope (S4300SE Hitachi, Tokyo, Japan). The CL signal was dispersed by a 1,200-lines/mm grating blazed at 500 nm, and CL spectra and images were recorded using a Peltier-cooled Hamamatsu R943-02 photomultipler tube.

## Results and discussion

### Structural characterization

The chemical composition of the synthesized nanocrystals measured by EPMA was Lu_0.990_Er_0.520_Yb_0.490_O_3_. The crystalline phase and unit cell parameters of the (Er,Yb):Lu_2_O_3_ nanocrystals are cubic with $\mathrm{Ia}\bar{3}$ space group and are reported in Table 1. FULLPROF software was used to refine the (Er,Yb):Lu_2_O_3_ nanocrystals and thus determine their lattice parameters (Table 1). As expected, the unit cell parameters increased by the introduction of Er^3+^ and Yb^3+^ to the matrix (erbium and ytterbium ions are larger than lutetium ion: ionic radii, Lu^3+^, cn = 6, 0.861 Å; ionic radii, Er^3+^, cn = 6, 0.890 Å; ionic radii, Yb^3+^, cn = 6, 0.868 Å [25]). In addition, Scherrer's equation was used to estimate a particle size of about 14.9 nm.

**Table 1:** **Unit cell parameters of (Er,Yb):Lu**_**2**_**O**_**3 **_**nanocrystals and of undoped Lu**_**2**_**O**_**3**_**, Er**_**2**_**O**_**3**_, **and Yb**_**2**_**O**_**3 **_**as reference**

**Stoichiometric formula**^**a**^	**Active ion (at.%)**		***a *(Å)**	***V *(Å**^**3**^**)**	**Particle size (nm)**^**b**^
	**Er**	**Yb**			
Lu_2_O_3_^c^			10.39	1,121.62	
Lu_0.990_ Er_0.520_ Yb_0.490_O_3_	25	25	10.4417 (4)	1,138.45(8)	14.9
Er_2_O_3_^d^			10.54800	1,173.57	
Yb_2_O_3_^e^			10.43470	1,136.16	

^a^Measured by EPMA; ^b^calculated using Scherrer's equation; ^c^JCPDS Lu_2_O_3_ (43–1021); ^d^JCPDS Er_2_O_3_ (43–1007); ^e^JCPDS Yb_2_O_3_ (41–1106).

**Figure 2:**
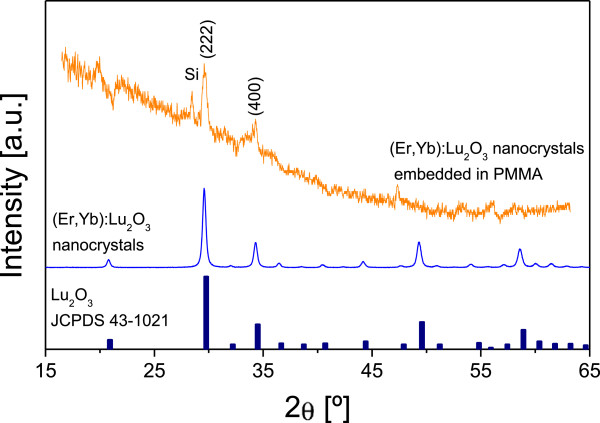
**XRD pattern of (Er,Yb):Lu**_**2**_**O**_**3 **_**immersed in PMMA and (Er,Yb):Lu**_**2**_**O**_**3 **_**nanocrystals and JCPDS 43–1021 as reference pattern.**

These nanocrystals embedded in the PMMA matrix were structurally characterized by μXRD, which made it possible to examine very small sample areas. We observed that the (Er,Yb):Lu_2_O_3_ nanocrystals embedded in PMMA microcolumns presented the two main diffraction peaks attributed to the cubic system with the $\mathrm{Ia}\bar{3}$ space group (Figure 2) and some extra peaks of the silicon mask. As expected, no preferential orientation was shown in the nanocrystals embedded in the PMMA columns.

### Particle size and dispersion

**Figure 3:**
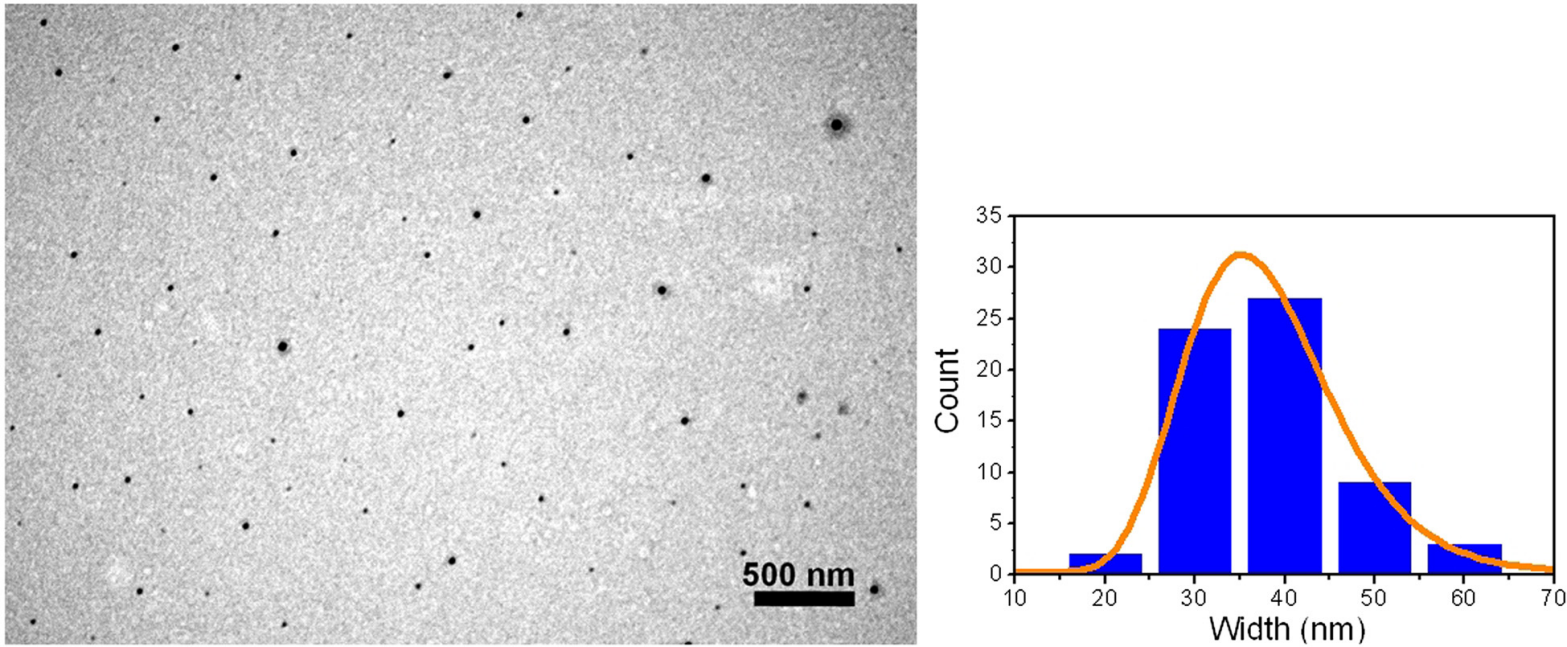
**TEM image of the (Er,Yb):Lu**_**2**_**O**_**3 **_**nanocrystals.**

**Figure 4:**
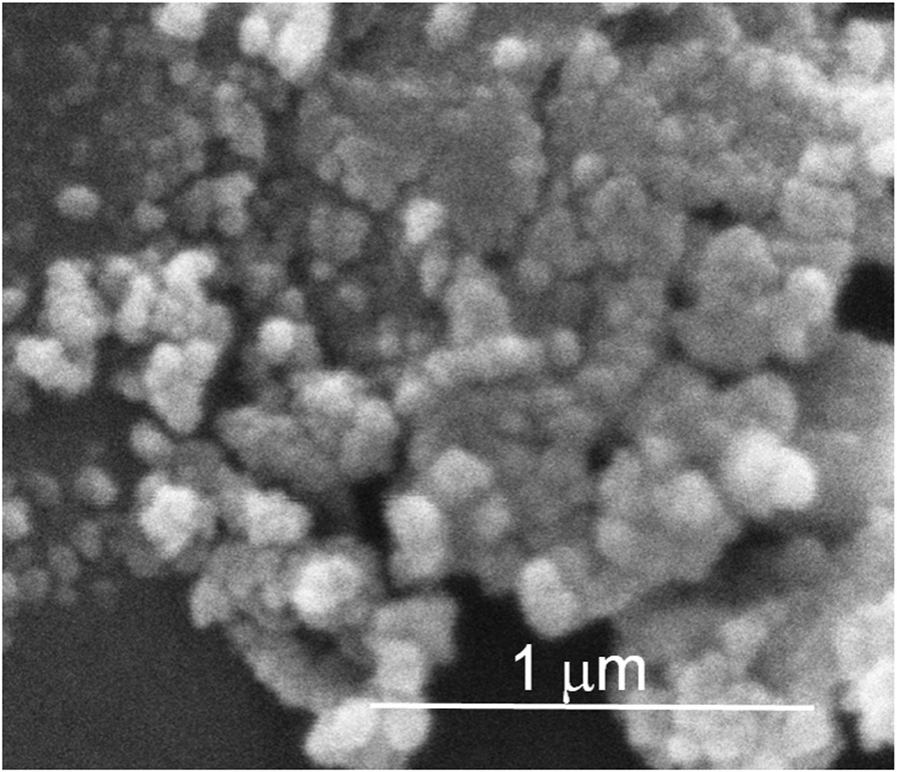
**ESEM image of the (Er,Yb):Lu**_**2**_**O**_**3 **_**nanocrystals.**

The particle size and dispersion were studied using TEM imaging and software. Figure 3 shows the representative TEM images and the histogram of the (Er,Yb):Lu_2_O_3_ nanocrystals, which is well represented by a lognormal distribution with a mean size of 33.1 nm and a dispersion of 44% [26,27]. Moreover, the sample presents good homogeneity, but the nanocrystals build aggregates that lead to large particle size dispersion (Figure 4). As reported in our other previous works, we can observe an almost spherical morphology of the nanocrystals, which is related with the polyhedrical shape of the nanocrystals. Using the Wulff theory and Donnay-Harker theory [28], in which the morphological importance of the crystalline faces is proportional to 1/*d*_hkl_; we can say that the crystalline habit in (Er,Yb):Lu_2_O_3_ nanocrystals is dominated by the crystallographic planes {2 0 0} and {1 1 2}.

### Visualization of PMMA microcolumns by electron microscopy

**Figure 5:**
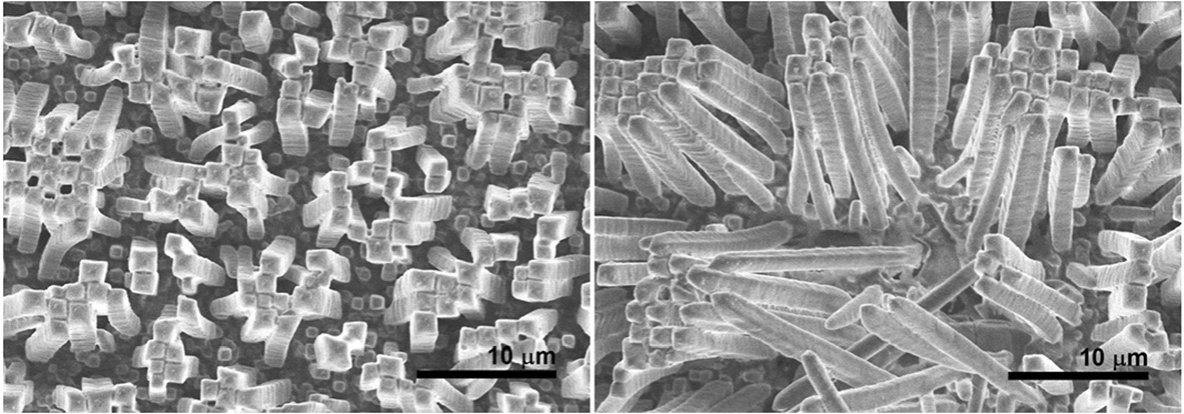
**ESEM images of PMMA microcolumns with embedded (Er,Yb):Lu**_**2**_**O**_**3 **_**nanocrystals.**

**Figure 6:**
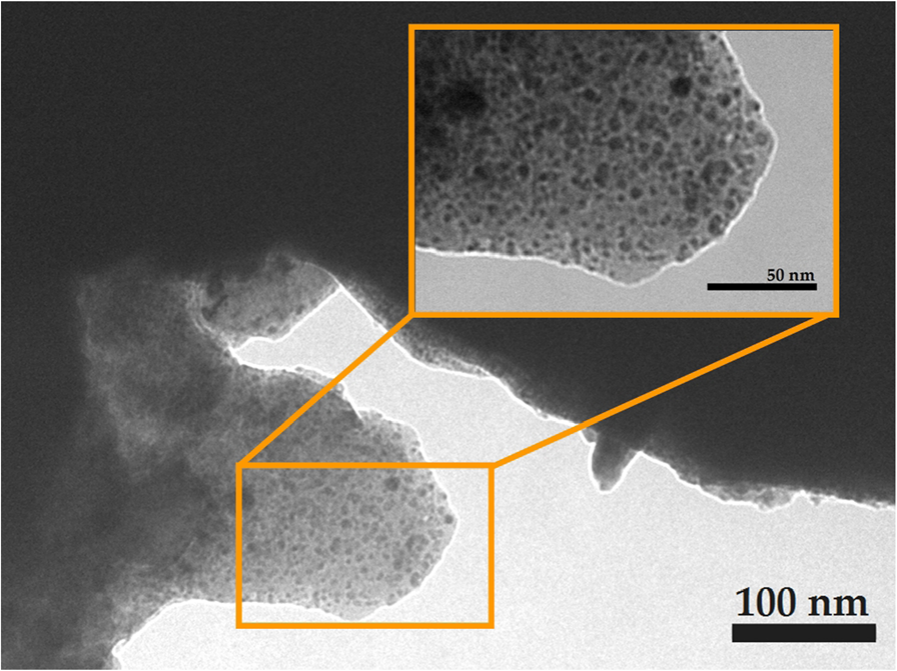
**TEM photographs of a fragment of PMMA microcolumns in which (Er,Yb):Lu**_**2**_**O**_**3 **_**nanocrystals are embedded.**

Environmental scanning electron microscopy was used to visualize the PMMA microcolumns after the silicon template had been removed (Figure 5). It can be observed that the microcolumns were disordered because they were grown on a disordered silicon template. The diameter of the microcolumns and the length of the columns were about 1 and 15 μm, respectively, resulting in an aspect ratio (height/diameter) of around 15. It was difficult to visualize the (Er,Yb):Lu_2_O_3_ nanocrystals in the microcolumns using ESEM, so transmission electron microscopy was used instead. Figure 6 shows some TEM images of a piece of PMMA microcolumn and shows the (Er,Yb):Lu_2_O_3_ nanocrystals with a darker contrast distributed in the microcolumns.

**Figure 7:**
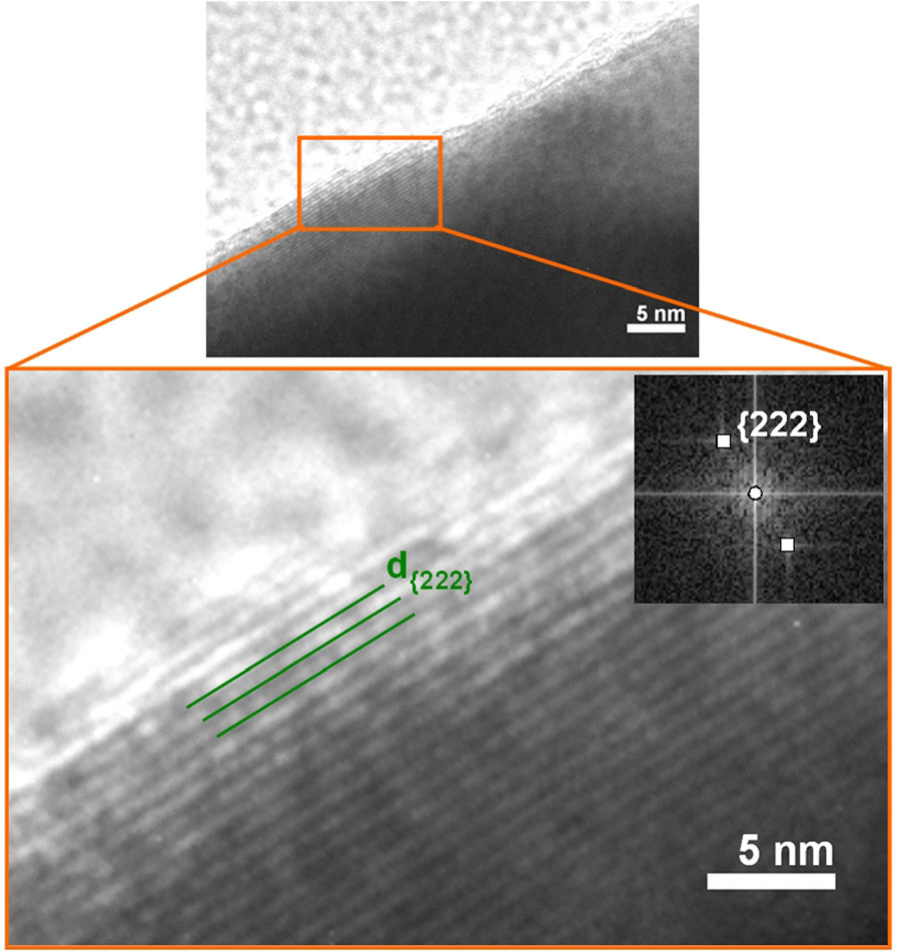
**HRTEM image and FFT pattern of (Er,Yb):Lu**_**2**_**O**_**3 **_**nanocrystals immersed in PMMA microcolumns.**

High-resolution electron microscopy was used to observe the (Er,Yb):Lu_2_O_3_ nanocrystals embedded in the PMMA microcolumns (Figure 7). The HRTEM images with the corresponding fast Fourier transform (FFT) pattern and the lattice planes can be indexed on the basis of their cubic phase. A border of nanocrystals clearly shows an interplanar {2 2 2} lattice with a value of 3.014 Å, and this was also confirmed by an FFT pattern indicating the high crystallinity degree of the nanocrystals.

### Cathodoluminescence measurements

**Figure 8:**
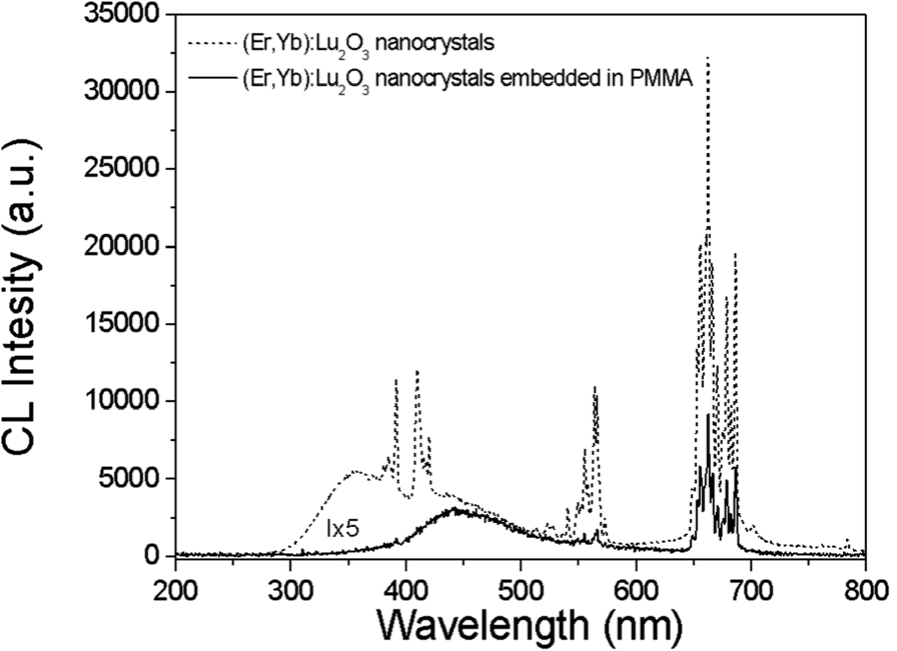
**Cathodoluminescence spectra of (Er,Yb):Lu**_**2**_**O**_**3 **_**nanocrystals and (Er,Yb):Lu**_**2**_**O**_**3 **_**nanocrystals embedded into PMMA microcolumns.**

We investigated the cathodoluminescence of (Er,Yb):Lu_2_O_3_ nanocrystals in air and embedded in the PMMA microcolumns in the visible range (see Figure 8, which also shows the f-f transitions of Er^3+^ assignment). The excitation voltage used was 15 kV and the probe current was about 10 nA.

As in the work of Yang et al. [29], the electron penetration depth, *L*_p_, can be estimated using the expression *L*_p_ = 250 (MW / *ρ*)(*E*/*Z*^1/2^)*n*, where *n* = 1.2(1 to 0.29 log_10_*Z*), MW is the molecular weight of the material, *ρ* is the bulk density, *Z* is the atomic number, and *E* is the accelerating voltage (kV). The deeper the electrons penetrate the phosphor, the greater the increase in the electron-solid interaction volume and consequently in the quantity of Ln^3+^ excited ions. Using this approach, our penetration depth was estimated to be about 18 μm. This would correspond to the total height of the PMMA microcolumns.

Four manifolds were mainly observed, and these correspond to the following electronic transitions: ^4^G_11/2_ → ^4^I_15/2_ (violet emission centered on 380 nm), ^2^H_9/2_ → ^4^I_15/2_ (blue emission centered around 410 nm), ^4^S_3/2_ → ^4^I_15/2_ (green emission centered on 560 nm), and finally ^4^F_9/2_ → ^4^I_15/2_ (red emission centered on 680 nm). Broad band emission acting as a background is observed centered around 400 nm. A similar broad band which has been attributed to radiative recombination at defect centers has been also detected by cathodoluminescence in previous works [30,31]. It could be observed that the intensity of the peaks decreases when the nanocrystals are embedded in the polymer matrix; therefore, only the last two transitions can be observed in these spectra. This fact could be attributed to the less quantity of the optical active material and to some scattering in the PMMA columns as a result of their apparent roughness.

As reported in previous works [32,33], the red emission (Er^3+^: ^4^F_9/2_ → ^4^I_15/2_) was observed to predominate over the green emission (Er^3+^: (^2^H_11/2_, ^4^S_3/2_) → ^4^I_15/2_). This has been related to a ^4^I_11/2_ → ^4^I_13/2_ large nonradiative relaxation rate with a ^4^F_9/2_ → ^4^I_9/2_ small nonradiative relaxation rate, and this relation with the large ^4^I_11/2_ → ^4^I_13/2_ nonradiative relaxation rate is attributed to the occurrence of an efficient cross energy transfer to the OH− surface group as a result of the good energy match.

Furthermore, it was proposed that a cross-relaxation process was responsible for populating the ^4^F_9/2_ level and that this occurs via two resonant transitions: ^4^F_7/2_ → ^4^F_9/2_ and ^4^F_9/2_ → ^4^I_11/2_. In fact, the cross-relaxation process becomes more efficient as the average distance between the doping ions decreases and, therefore, the Er^3+^ concentration increases, thus enhancing the red emission. Our samples possess a 25 at.% erbium concentration, which is higher than the concentrations reported in previous studies [33]. This also agrees well with the results of Yang et al. [29], who observed the predominance of green emission and the absence of red emission in flower microcrystallites that had been low doped with 1 at.% Er:Lu_2_O_3_.

Furthermore, as it can be observed in Figure 8, there is a change on the blue/green/red emission ratio when the nanocrystals are embedded in the PMMA. This change could be related to a change in the up-conversion mechanism affected by the presence of the high-energy phonons of the polymer, favoring the red emission in relation to the green emission which has decreased and the blue emission which has totally disappeared.

**Figure 9:**
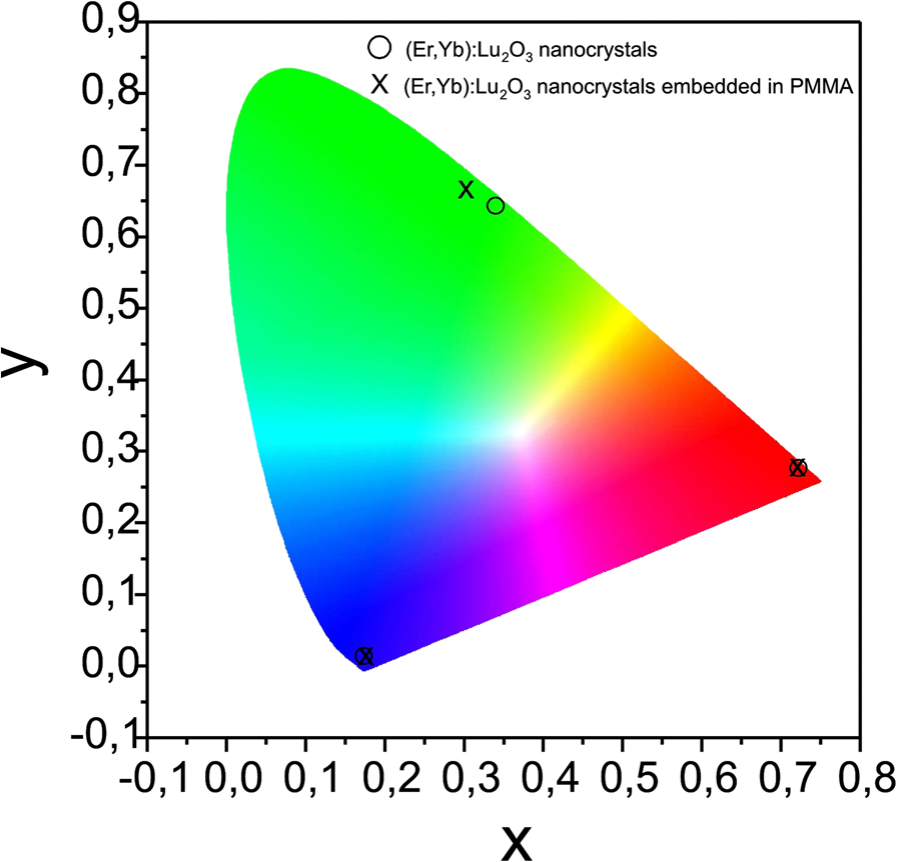
**CIE chromaticity diagram showing the emission colors for (Er,Yb):Lu**_**2**_**O**_**3 **_**and (Er,Yb):Lu**_**2**_**O**_**3 **_**nanocrystals embedded in PMMA microcolumns.**

For lighting applications, it is interesting to calculate the different parameters, which characterizes the color of the emission (see Table 2). The International Commission on Illumination (CIE) coordinates (*x*, *y*) specify where the point corresponding to each emission is located on the chromaticity diagram. In this diagram, the color of the light emitted is factored by the sensitivity curves measured for the human eye (color matching functions) (Figure 9). The dominant wavelength is the point of interception in the spectrum locus for the line crossing the white point and the point of each emission, and the purity is the saturation of a particular color. The greater the purity, the more saturated the color appears, that is, the more similar the color is to its spectrally pure color at the dominant wavelength. The values in Table 2 show that embedding the nanocrystals inside the PMMA matrix does not strongly affect their colorimetric properties. Furthermore, the red emission has the greatest purity and therefore the most saturated color.

**Table 2:** **Summary of CIE properties of (Er,Yb):Lu**_**2**_**O**_**3 **_**nanocrystals and (Er,Yb):Lu**_**2**_**O**_**3 **_**nanocrystals embedded in PMMA microcolumns**

	**Blue emission**				**Green emission**				**Red emission**			
	***x***	***y***	**Purity**	**Dominant wavelength**	***x***	***y***	**Purity**	**Dominant wavelength**	***x***	***y***	**Purity**	**Dominant wavelength**
			**(%)**	**(nm)**			**(%)**	**(nm)**			**(%)**	**(nm)**
(Er,Yb):Lu_2_O_3_ nanocrystals	0.1746	0.0137	97	375	0.3402	0.6423	96	556	0.7222	0.2777	100	643
(Er,Yb):Lu_2_O_3_ nanocrystals embedded in PMMA	0.1753	0.0132	97	362	0.3016	0.6661	92	550-554	0.7209	0.2789	99	642

## Conclusions

The modified Pechini method was successfully applied to obtain cubic nanocrystals of Lu_0.990_Er_0.520_Yb_0.490_O_3_. Scherrer's approach and electronic microscopy gave us an average size of about 15 to 30 nm with 44% dispersion size. The (Er,Yb):Lu_2_O_3_ nanocrystals were embedded in PMMA microcolumns prepared by vacuum infiltration. The PMMA columns solidified inside the micropores of a silicon matrix to form 2D disordered arrays. The embedded nanocrystals showed a high degree of crystallinity, and their cathodoluminescence properties were not altered by the polymer matrix. It is interesting to point out how the erbium red emission, with its dominant wavelength of 642 nm, its CIE coordinates (0.72, 0.28), and its high color saturation, predominates over the visible emission. This is attributed to the high erbium concentration present in the samples.

## Competing interests

The authors declare that they have no competing interests.

## Authors’ contributions

MG performed all experimental work, interpreted the data, and wrote the manuscript. MCP and JJC contributed to the concept of the study and revised the manuscript. XM participated in the interpretation of data and revised the manuscript. PF contributed to the design of the study and performed the preparation of composites. KHP, FR, and KK realized CL experiments and their interpretation. JP, LFM, MA, and FD revised critically the manuscript. All authors read and approved the final manuscript.
